# Comparative proteome analysis of human epithelial ovarian cancer

**DOI:** 10.1186/1477-5956-5-16

**Published:** 2007-09-24

**Authors:** Jean-Philippe Gagné, Chantal Éthier, Pierre Gagné, Geneviève Mercier, Marie-Ève Bonicalzi, Anne-Marie Mes-Masson, Arnaud Droit, Eric Winstall, Maxim Isabelle, Guy G Poirier

**Affiliations:** 1 Health and Environment Unit, Laval University Medical Research Center, CHUQ, Faculty of Medicine, Laval University, 2705, Boulevard Laurier, Ste-Foy, Québec, G1V 4G2, Canada; 2 Proteomics Platform, Québec Genomic Center, Laval University Medical Research Center, CHUQ, Faculty of Medicine, Laval University, 2705, Boulevard Laurier, Ste-Foy, Québec, G1V 4G2, Canada; 3 Centre de recherche du Centre Hospitalier de l'Université de Montréal (CHUM)-Hôpital Notre-Dame and Institut du cancer de Montréal, 1560 rue Sherbrooke Est, Montréal, Québec, H2L 4M1, Canada; 4 CNRS UMR6061 Université de Rennes 1, Groupe Cycle Cellulaire, Université de Rennes 1, Faculté de Médecine, 2 Avenue du Pr Léon Bernard, CS 3417, Rennes cedex, France

## Abstract

**Background:**

Epithelial ovarian cancer is a devastating disease associated with low survival prognosis mainly because of the lack of early detection markers and the asymptomatic nature of the cancer until late stage. Using two complementary proteomics approaches, a differential protein expression profile was carried out between low and highly transformed epithelial ovarian cancer cell lines which realistically mimic the phenotypic changes observed during evolution of a tumour metastasis. This investigation was aimed at a better understanding of the molecular mechanisms underlying differentiation, proliferation and neoplastic progression of ovarian cancer.

**Results:**

The quantitative profiling of epithelial ovarian cancer model cell lines TOV-81D and TOV-112D generated using iTRAQ analysis and two-dimensional electrophoresis coupled to liquid chromatography tandem mass spectrometry revealed some proteins with altered expression levels. Several of these proteins have been the object of interest in cancer research but others were unrecognized as differentially expressed in a context of ovarian cancer. Among these, series of proteins involved in transcriptional activity, cellular metabolism, cell adhesion or motility and cytoskeleton organization were identified, suggesting their possible role in the emergence of oncogenic pathways leading to aggressive cellular behavior.

**Conclusion:**

The differential protein expression profile generated by the two proteomics approaches combined to complementary characterizations studies will open the way to more exhaustive and systematic representation of the disease and will provide valuable information that may be helpful to uncover the molecular mechanisms related to epithelial ovarian cancer.

## Background

Despite years of research in clinical aspects of ovarian cancer, this gynaecological pathology is still one of the most deadly cancers among women in most western countries. The search for biomarkers to detect early phase ovarian cancer and to monitor disease progression has been targeted for a long time by the medical community. DNA microarray expression profiling-based research was applied to identify candidate genes that may account for tumorigenesis as well as proteomics-based search for specific protein biomarkers that could facilitate the detection of ovarian cancer. However, few quantitative systematic analysis of ovarian cancer by proteomics approaches have been undertaken. Recently, we reported a proteome profiling of the TOV-112D cell line, a human model for the study of epithelial ovarian cancer [1]. To go further in the comprehension of ovarian carcinogenesis, we have investigated the differential protein expression profile between low malignant potential and highly proliferative human epithelial ovarian cancer cell lines TOV-81D and TOV-112D. These cell lines are spontaneously immortalized epithelial ovarian cancer cell lines derived from ovarian malignant tumours [2]. The growth characteristics and tumorigenic potential of these cell lines parallel the prognosis of the patients from which these cell lines are derived. The TOV-112D cell line comes from an extremely aggressive ovarian endometrioid tumor (grade 3) while the TOV-81D originates from an intermediate grade (grade 1–2) but a clinically rather indolent papillary serous adenocarcinoma. The TOV-81D cell line has been the subject of several microarray-based analysis that revealed a high similarity to normal ovarian surface epithelium [3-5], a distinctive feature that suggests that it is an excellent baseline for comparisons. Ovarian cancers are characterized by extensive molecular alterations and complex chromosomal aberrations. However, normal ovarian surface epithelium and TOV-81D cell line display few chromosomal differences, an additional feature that makes this cell line a good model in a differential profile framework [2]. The morphology of TOV-81D cells is highly similar to the morphology of normal ovarian epithelium, in opposition to TOV-112D cells that are smaller and more refractile, a characteristic of highly transformed cell lines. TOV-112D provides several useful advantages for our specific study. In particular, it rates amongst the most aggressive EOC cell lines and has the added advantage that it was derived from a chemotherapy naïve patient, a condition that minimize genetic alterations often associated with adjuvant therapy such as chemotherapy or radiation therapy [2] and thus it might be argued that is more closely recapitulates the fundamental molecular changes associated with ovarian cancer. Although the histopathology of these two cell lines may appear diverse, there is a growing body of literature that suggests that both from a molecular [6] and pathological (reviewed in Gilks, 2004 [7]) point of view that it is doubtful whether there is a consistently recognizable set of high-grade endometrioid carcinomas that differ in any substantive way from high-grade papillary serous carcinomas. Both TOV-81D and TOV-112D have been extensively characterized at a genomic and transcriptomic levels and therefore make this model particularly attractive from a systems biology point of view [3-5,8-10]. Taking into account all the pertinent characteristics of these cell lines supports the notion that the differential protein expression analysis of TOV-81D and TOV-112D cell lines provides an attractive model to assess molecular events associated with EOC.

Two quantitative proteomics approaches were selected for comparing TOV-81D and TOV-112D proteomics profiles: isobaric tags for relative and absolute quantitation (iTRAQ) analysis and two-dimensional electrophoresis (2DE) coupled to liquid chromatography tandem mass spectrometry (LC MS/MS). The first approach is a gel free mass spectrometry technique that uses isobaric amine specific tags to compare the peptide intensities between samples and infer quantitative values for corresponding proteins. The second approach is based on the differential two-dimensional gel electrophoresis pattern between protein samples. This alternative approach gives addition biological information such as isoelectric point drift or molecular weight alterations from which we can suggest important implications to protein functions. Both methods generate quantitative data that provide a differential protein dataset when a protein expression ratio between TOV-81D and TOV-112D cell lines is applied. These complementary technologies [11] reinforces the identification of distinctive protein expression between TOV-81D and TOV-112D and provide a reliable tool to estimate relevant protein changes in the context of human ovarian cancer. This study is dedicated to explore the proteome's molecular alterations associated with ovarian cancer, providing helpful information that could be used in conjuction with complememtary approaches such as gene expression profiling to have a more inclusive and global view of the disease.

## Results and discussion

### Comparative iTRAQ™ protein expression analysis between TOV-81D and TOV-112D epithelial ovarian cancer model cell lines

#### The Gene Ontology classification of differentially expressed proteins reveals a clusterizing of proteins

**Figure 1:**
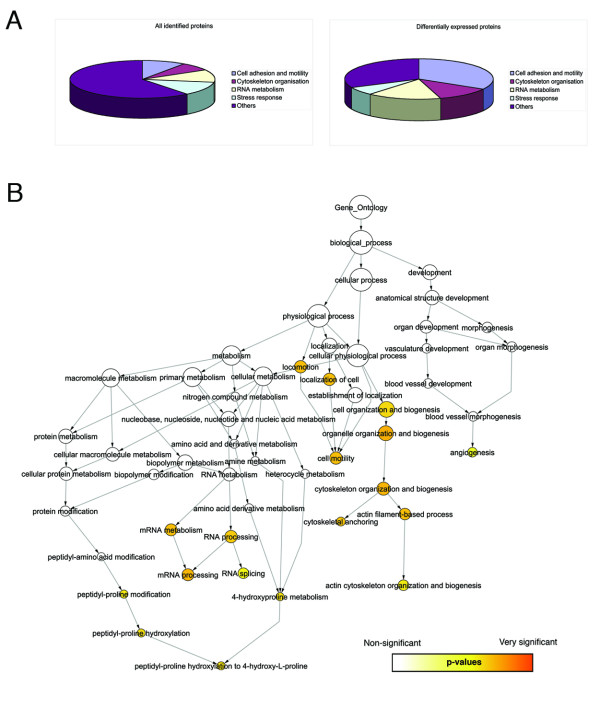
Distribution of the proteins identified by iTRAQ analysis according to Gene Ontology (GO) categories. (**A**) Relative distribution of proteins in selected GO terms for proteins that meet the under- or overexpression threshold for iTRAQ ratios between TOV-81D and TOV-112D cell lines compared to the overall protein identifications. (**B**) BiNGO determination of statistically overrepresented GO categories for the differentially expressed proteins between TOV-81D and TOV-112D cell lines. The biological network subgraph has been visualized using Cytoscape software.

The quantitative evaluation of protein expression profiles between low and high malignancy ovarian cancer cell lines was performed using the iTRAQ technology which infer relative protein abundance from MS analysis [12]. The threshold for potentially significant change in protein expression has been statistically determined using Statgraphics Centurion's (version 15.1.03, StatPoint, Herndon, VA) generalized logistic distribution to the ratios (TOV112D/TOV81D) computed for all the iTRAQ identified proteins [see Additional file-1]. At a p-value of 0.10 (90% confidence), ratios above 2.5 and under 0.59 are significantly different from the average to be considered as a potential change in protein expression. At a p-value of 0.05, these values are 3.0 and 0.41. Values at 90% confidence were used as a cut-off in this study [see Additional file-2] although ratios at 95% confidence should be kept in mind for more confident change in protein expression. The most prominent protein expression changes are listed in Table 1 as 37 differentially expressed proteins that meet the 95% confidence threshold criteria. A biological process clustering of these proteins based on the Gene Ontology (GO) Consortium [13] annotations was created in order to perceive the distribution of the differentially expressed proteins within important cellular regulatory functions (Figure 1). Proteomics studies generate large amounts of data that needs to be structured for easier interpretation and evaluation of biological relevance. Ontologies provide such structured description of biological information that can be further analyzed into clusters of functionally related proteins. In the present study, rather than following the distribution trend associated with all the iTRAQ identifications, over- and underexpressed proteins are enriched in specific GO categories. Notably, proteins involved in cytosqueleton organization, cell motility and adhesion are over-representated relative to their proportion in the overall protein population (Figure 1A). This concept has been extended by assessing statistical overrepresentation of specific GO categories using BiNGO, a tool developped to highlight predominant functional themes in a dataset and to visualize them as an integrated molecular interaction network. (Figure 1B).

**Table 1:** iTRAQ analysis of differentially expressed proteins between human epithelial ovarian cancer cell lines TOV-112D and TOV-81D†.

Accession	Entrez Gene	GO	Gene	SwissProt	Description	Number of unique peptides	Protein ratio (112D/81D)	%RSD	Validation
IPI00008557	10642	RNA metabolism/protein biosynthesis	IGF2BP1	Q9NZI8	Insulin-like growth factor 2 mRNA-binding protein 1	2	11,9	81	
IPI00005996	8091	Transcription	HMGA2	P52926	High mobility group protein HMGI-C	2	8,1	100	
IPI00218914	216	Response to stress	ALDH1A1	P00352	Aldehyde dehydrogenase 1A1	6	5,8	43	2D
IPI00021033	1281	Cell adhesion	COL3A1	P02461	Collagen alpha-1(III) chain precursor	4	5,0	79	2D
IPI00014424	1917	Protein biosynthesis	EEF1A2	Q05639	Elongation factor 1-alpha 2	3	4,2	80	2D
IPI00028376	1678	Transport/protein metabolism	TIMM8A	O60220	Mitochondrial import inner membrane translocase subunit Tim8A	2	4,2	14	
IPI00030131	7112	Cytoskeleton organizationanisation	TMPO	P42167	Lamina-associated polypeptide 2, isoforms beta/gamma	3	3,8	21	
IPI00329745	10128	RNA metabolism	LRPPRC	P42704	130 kDa leucine-rich protein	3	3,8	30	
IPI00301189	51477	Lipid biosynthesis	ISYNA1	Q9NPH2	Myo-inositol 1-phosphate synthase A1	2	3,7	23	
IPI00165467	10643	RNA metabolism/protein biosynthesis	IGF2BP3	O00425	Insulin-like growth factor 2 mRNA-binding protein 3	3	3,3	50	
IPI00292387	9221	RNA metabolism	NOLC1	Q14978	Nucleolar phosphoprotein p130	2	3,3	22	
IPI00218493	3251	Puridine salvage	HPRT1	P00492	Hypoxanthine-guanine phosphoribosyltransferase	2	3,1	15	
IPI00218918	301	Membranes binding/cell motility	ANXA1	P04083	Annexin A1	7	3,0	41	2D&WB
IPI00216044	22913	RNA metabolism	RALY	Q9UKM9	RNA-binding protein Raly	2	3,0	18	
IPI00000105	9961	Response to stress	MVP	Q14764	Major vault protein	3	0,40	87	
IPI00455315	302	Membranes binding/cell motility	ANXA2	P07355	Annexin A2	3	0,40	33	
IPI00477536	2317	Cell motility/Cytoskeleton organization/signal transduction	FLNB	Q60FE7	Filamin B	2	0,38	47	
IPI00333541	2316	Cell motility/Cytoskeleton organization/signal transduction	FLNA	P21333	Filamin-A	10	0,36	46	
IPI00296099	7057	Cell motility/cell adhesion	THBS1	P07996	Thrombospondin-1 precursor	2	0,35	67	
IPI00013808	81	Cell motility/cell adhesion	ACTN4	O43707	Alpha-actinin-4	2	0,33	22	
IPI00029111	1809	Pyrimidine metabolism	DPYSL3	Q6DEN2	Dihydropyrimidinase-like 3 protein (DPYSL3 protein)	4	0,30	85	2D
IPI00554788	3875	Cytoskeleton organizationanisation	KRT18	P05783	Keratin-18	2	0,29	19	
IPI00306604	3678	Cell adhesion	ITGA5	P08648	Integrin alpha-5 precursor	2	0,29	45	
IPI00163187	6624	Cytoskeleton organization/cell motility	FSCN1	Q16658	Fascin	2	0,24	18	2D
IPI00031008	3371	Cell adhesion	TNC	P24821	Tenascin-C	2	0,23	71	
IPI00182373	8974	Collagen biosynthesis	P4HA2	O15460	Prolyl 4-hydroxylase subunit alpha-2 precursor	5	0,22	22	2D
IPI00472165	5352	Collagen biosynthesis	PLOD2	O00469	Procollagen-lysine,2-oxoglutarate 5-dioxygenase 2 precursor;	3	0,21	55	2D
IPI00550363	8407	Cytoskeleton organization/cell motility	TAGLN2	P37802	Transgelin-2	2	0,20	11	2D&WB
IPI00216138	6876	Cytoskeleton organization/cell motility	TAGLN	Q01995	Transgelin	5	0,20	58	
IPI00008494	3383	Cell adhesion	ICAM1	P05362	Intercellular adhesion molecule 1 precursor (ICAM1)	4	0,17	35	
IPI00216135	7168	Cell motility/cell adhesion	TPM1	P09493	Tropomyosin alpha-1 chain	2	0,16	38	2D
IPI00007118	5054	Cell adhesion/motility/urokinase regulation	SERPINE1	P05121	Plasminogen activator inhibitor 1 precursor (PAI-1)	2	0,15	27	2D
IPI00414283	2335	Cell motility/cell adhesion	FN1	P02751	Fibronectin precursor	2	0,11	8	
IPI00026663	220	Response to stress	ALDH1A3	P47895	Aldehyde dehydrogenase 1A3	3	0,11	39	2D
IPI00442073	1465	Cell motility	CSRP1	P21291	Cysteine and glycine-rich protein 1	3	0,08	255	
IPI00297646	1277	Cell adhesion	COL1A1	P02452	Collagen alpha-1(I) chain precursor	5	0,08	50	
IPI00178352	2318	Cell motility/Cytoskeleton organization/signal transduction	FLNC	Q14315	Filamin-C	3	0,07	39	

†Selected protein expression ratios were confirmed by western blot (WB) analysis. Differentially expressed proteins also identified by comparative two-dimensional electrophoresis are indicated (2D). %RSD = (standard deviation/mean) × 100

#### Differentially expressed proteins in the context of ovarian cancer

Several proteins identified in this study with strong iTRAQ differential expression ratios were previously studied in association with human ovarian cancer or other epithelial disorders, an indication that this study is actually addressing targets of interest. For example, The IGF II mRNA-binding protein 1 (formely Coding region determinant-binding protein (CRD-BP/IMP1)) which is characterized with the highest iTRAQ protein ratio have been proposed as a prognostic marker for patients with ovarian [14] and colon cancer [15]. This protein stabilizes the c-Myc protoncogene mRNA regions of instability which results in the protection of the messenger from endonucleolytic attack and thereby prolongs the mRNA half-life [16]. The levels of c-Myc protein has been linked to cell proliferation, differentiation and neoplastic transformation [17].

Another example is the high mobility group protein HMGI-C, also featuring a high differential expression ratio. HMG proteins are a family of architectural transcription factors establishing transcriptionaly active or inactive chromatin domains [18]. The HMGI-C gene is probably one of the most commonly rearranged gene in malignant tumours [19] so its identification as a highly overexpressed protein in TOV-112D is not surprising. The expression of high mobility group proteins have been evaluated in ovarian carcinomas and characterized as a frequent feature of ovarian cancer [20-22]. The latter proteins are interacting selectively with nucleic acid but other proteins involved in the metabolism of nucleic acid are also strongly enhanced in the TOV-112D protein expression profile compared to TOV-81D. One of these is the elongation factor 1-alpha 2 (eEF1A-2). It has been shown to be an oncogenic factor likely associated with the development of ovarian cancer [23] and also have been identified as a useful diagnostic marker and therapeutic target for a high proportion of breast tumours [24].

Differential gene expression analysis have been performed to understand the biochemical and molecular changes involved in the formation and progression of ovarian tumors. Notably, the most prominent overexpressed gene identified between advanced and local ovarian adenocarcinoma is collagen alpha 1(III) chain precursor (COL3A1) a gene which corresponding protein have been shown by our iTRAQ analysis to be overexpressed in TOV-112D. Interestingly, the product of the COL3A1 gene have been found to increase the degree of malignancy in serous ovarian carcinoma [25].

On the other hand, underexpressed proteins characterized with low iTRAQ ratio such as the transgelins were also described as important factors in early events involved in tumor progression and have been proposed as diagnostic markers for epithelial breast [26] and colon cancer [26,27]. The down regulation of transgelins in malignant cells is observed in various carcinomas including ovarian epithelial cancer [28]. Another example is thrombospondin-1 which as emerged as a protein with reduced expression in TOV-112D. Thrombospondin-1 is an angiogenesis inhibitor [29,30] that as been implicated in tumor growth and progression [31]. The reduction of thrombospondin expression has been suggested to result in the development of a pro-angiogenic environment and malignant phenotype in epithelial ovarian carcinoma [32]. The thrombospondin gene has also been identified as an underexpressed gene in a comparative gene expression analysis between three-dimensional epithelial ovarian cancer cultures and monolayers, an indication of potentially altered angiogenic signaling resulting from the reorganisation of matrix proteins and cell-surface receptors to which thrombospondin-1 interacts at the extracellular matrix [33].

Theses differentially expressed proteins were recognized in differential expression analysis and further investigated for their relationship with disease mechanisms. We believe that other targets revealed by the present study could provide usefull information for studies on ovarian cancer.

#### Downregulation of TOV-112D proteins involved in cell adhesion, motility and cytoskeleton organization

**Figure 2:**
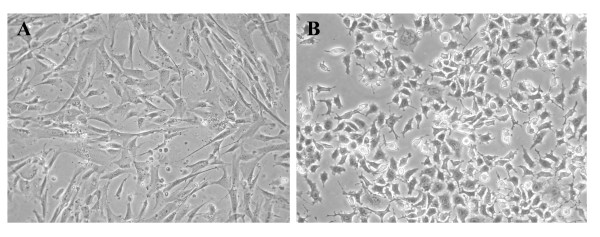
Morphological aspect of the TOV cell lines as observed under a phase contrast microscope. TOV-81D cells show a flat morphology similar to normal ovarian epithelium (A) while TOV-112D cells show a highly rounded morphology characteristic of highly transformed cell lines (B) as already published [2].

Rearrangement of extracellular membrane proteins and cytoskeletal microfilaments induce major cellular morphological alterations in transformed cells. The action of cross-linking proteins is particularly important to potentiate the invasiveness of tumor cells. The differential expression of some membrane-associated proteins and proteins that could be associated with cytoskeletal organization was expected given the pronounced morphological differences between TOV-112D and TOV81D (Figure 2) and their different growth characteristics. TOV-81D cells are unable to grow without solid support while TOV-112D cells formed large dense cell foci in semisolid medium [2]. Moreover, the injection of TOV-112D cell-line in nude mice result in the rapid appearance of tumours while no tumours are seen after the injection of TOV-81D cell line. A significant part of these differential growth characteristics are likely to be the consequence of changes in plasma membrane properties and dynamics. The identification of the extracellular matrix component collagen alpha 1 is particularly relevant since it is not only involved in structural support of tissues but could also exhibit modulatory effect on malignant cell behaviour [34]. Besides, the divergent growth features between the two cell lines could also be explained by different processing of extra cellular stimuli. The TOV-112D overexpressed protein myo-inositol 1-phosphate synthetase-1 is likely involved in the synthesis of inositol phospholipids, a component of plasma membrane that convey extracellular signals from a variety of peptide signalling molecules into cells [35].

In opposition to proteins whose abundance is increased in TOV-112D cells, proteins identified with biological functions in association with cytosqueleton organization are characterized with relatively low TOV112D:TOV81D ratios, an indication of underexpression pattern. A good exemple is the identification of the cysteine and glycine-rich protein 1 (CSRP1) which is one of the most underexpressed protein relative to iTRAQ differential expression analysis ratios. Recently, a zebra fish homolog of CSRP1 have been shown to control cell morphology and other dynamic cell behaviors [36]. It is conceivable that the loss of CSRP1 could play a role in the remodeling of TOV-112D cytosqueleton.

In addition, the iTRAQ comparative proteome analysis underscores the underexpression of a cluster of actin-binding proteins that are involved in integrin-mediated biological responses. Actually, Filamin A/B/C, Talin, Alpha-actinin1/4, Fascin and Integrin alpha-5 could all be involved in the integrin family of cell adhesion molecules pathways [37-39]. The underexpression of important molecules involved in extracellular matrix adhesion is consistent with the behaviour of TOV-112D cells which produces tumours rapidly, an indication of high mobility and invasion power.

Changes at the plasma membrane are also underscored by the drastic underexpression of cellular matrix and cell adhesion proteins such as fibronectin, intergrin or the intercellular adhesion molecule-1 (ICAM-1). These proteins participate to cellular migration and invasion behaviour. Since TOV-112D are not only highly proliferative but also easily disseminates compared to TOV-81D cells, the downregulation of proteins involved in cell adhesion support the idea of modified extracellular matrix composition that could afford for the different dissemination potential observed between the two cell lines. The plasminogen activator inhibitor-1 precursors (PAI-1) also have functions in line with the idea of differential biological behaviour based on plasma membrane phenotypes. PAI-1 downregulates the plasminogen activator that degrades a range of extracellular basement membrane components [40]. Since the expression of the major plasminogen inhibitor is dramatically reduced in TOV-112D cell lines, it is tempting to speculate that overactivation of extracellular membrane remodelling contributes to the metastatic capacity of this highly proliferative cell line. However, this result contrasts with mRNA expression analysis of PAI-1 in epithelial ovarian cancer where PAI-1 mRNA levels where shown to be elevated [41], in opposition to protein quantitation using iTRAQ comparative analysis.

#### Specific expression of protein isoforms

Another interesting feature that can be underscored from the iTRAQ analysis is the importance of specific protein isoforms that characterized each cell lines. For example, the aldehyde dehydrogenase family 1 member A1 (ALDH1A1) is robustly overexpressed in TOV-112D compared to TOV-81D but the ALDH1A3 isoform expression is strongly repressed. ALDH1A1 and ALDH1A3 genes are closely related but distinct members of the aldehyde dehydrogenase genes superfamily which are important enzymes involved in the oxidative stress response and detoxification processes with a general expression increase in several tumours [42]. The same observation is applicable to type-III and type-I collagen precursors that are respectively overexpressed and underexpressed in TOV-112D cells when compared to TOV-81D cells. Indeed, type-III collagen has been suggested to play a role in breast cancer [43] and serous ovarian carcinoma [44]. There is also comparable discrepancy between annexin A1 and annexin A2, two related proteins with possible roles into membrane dynamics, cell differentiation and migration [45] that are characterized with highly divergent TOV112D:TOV81D expression ratios.

### Differential protein expression analysis between TOV-81D and TOV-112D cell lines by two-dimensional electrophoresis

In contrast to LC-based quantitative proteomics such as iTRAQ analysis, the gel-based two-dimensional electrophoresis is still very laborious although major technical improvements have been developed over recent years. 2DE is a powerful tool for comparative protein expression with complementary features over LC-based proteomics with meaningful contribution to the understanding of cell adaptation to tumour environment. The strength of 2DE lies in the direct visualisation of post-translational alterations that are reflected by changes in spot localizations.

**Figure 3:**
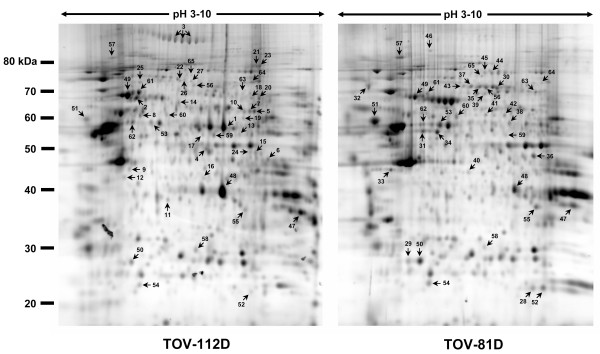
Representative 2DE gel image visualized by Sypro Ruby staining. 250 μg of each protein extract was loaded on an immobilised pH gradient strip (pH 3–10 non linear) followed by a 10 % SDS-PAGE. Spots corresponding to LC MS/MS identified proteins are numbered from differentially expressed proteins between TOV-112D and TOV-81D as given by Gaussian modeling with the PDQuest ™ software (referenced from Table 2).

**Figure 4:**
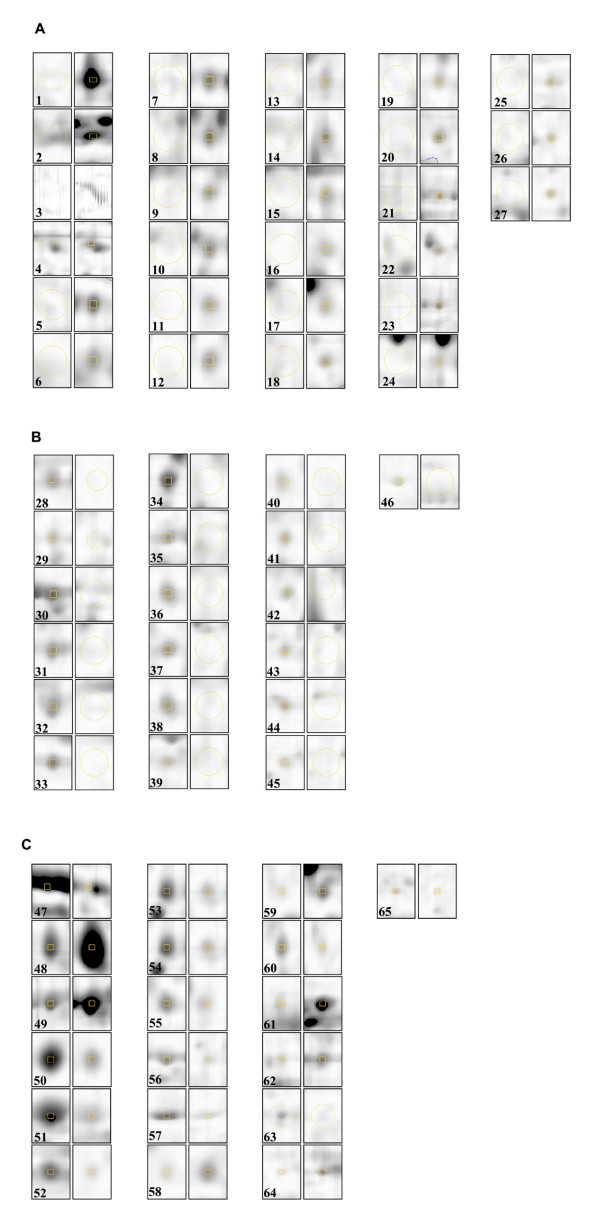
Zoomed sections from TOV-112D and TOV-81D gels demonstrating differential expression of proteins listed in Table 2. (A) Selected TOV-112D spots with undetectable TOV-81D matching spots. (B) Selected TOV-81D spots with undetectable TOV-112D matching spots. (C) Selected spots differentially expressed with a minimum deregulation ratio of 2.0.

A reliable comparative proteome analysis using 2DE-based analysis requires the use of replicate groups to overcome the inherent experimental variations that precluded the obtention of reproducible and quantitative datasets. Replicate group analysis ensures that consistent coordinates are acquired for every detected spots and make possible statistical analysis of the data (Figure 3). This approach resulted in the identification of several spots that differ consistently between the two cell lines. A selection of manually reviewed spots localized in well focused area of the gel that unambiguously meet both a two-fold expression threshold and a Student's parametric test assuming a normal distribution for small samples is presented in Figure 4 and the corresponding Table 2. In general, the correlation of protein expression dataset from 2DE follows the same trend as the one generated by iTRAQ analysis. For example, spots corresponding to collagen alpha (Spot #3) and aldehyde dehydrogenase (Spot#1) are unequivocally hallmarks of TOV-112D overexpressed proteins to which strong iTRAQ ratios were also determined. Inversely, very low iTRAQ protein ratios that indicate a dramatic decrease of TOV-112D protein expression compared to TOV-81D have been calculated for proteins such as prolyl 4-hydroxylase alpha-1 subunit precursor (Spot#60), procollagen-lysine, 2-oxoglutarate 5-dioxygenase (Spot#44–45), plasminogen activator inhibitor-1 precursor (Spot#36) or tropomyosin (Spot#32). All of these proteins were identified by 2DE to be overexpressed in TOV-81D. Together, the iTRAQ and 2DE datasets add confidence and credibility to the differential proteome analysis but the two methods are clearly complementary since the two datasets do not completely overlap. The identification of several 2DE spots with intense overexpression as heat shock 70-kDa protein (Spot#2) without equivalent iTRAQ ratios illustrate this complementarity that could be explained by the different physico-chemical processing of the samples or different peptide representation between the two methods [11].

**Table 2:** Comparative protein expression analysis of TOV-81D and TOV-112D cell lines by two-dimensional electrophoresis (see Figure 4 for spot review).

Selected TOV-112D spots with undetectable TOV-81D matching spots		
Spots positions	IPI Accession number	Description

1	IPI00218914.1	Aldehyde dehydrogenase 1A1
2	IPI00304925.1	Heat shock 70 kDa protein
3	IPI00021033.1	Collagen alpha 1(III) chain precursor
4	IPI00299000.1	Proliferation-associated protein 2G4
5	IPI00027834.2	Heterogeneous nuclear ribonucleoprotein L
6	IPI00025366.1	Citrate synthase, mitochondrial precursor
7	IPI00027834.2	Heterogeneous nuclear ribonucleoprotein L
8	IPI00302925.1	T-complex protein 1, theta subunit
9	IPI00164305.1	Membrane associated protein SLP-2
10	IPI00414123.1	Collapsin response mediator protein 1 (CRMP-1)
11	IPI00300086.1	Nicotinate-nucleotide pyrophosphorylase
12	IPI00008552.2	Thioredoxin-like protein 2
13	IPI00221234.1	Antiquitin
14	IPI00219077.1	Leukotriene A-4 hydrolase
15	IPI00001661.1	Regulator of chromosome condensation (RCC1)
16	IPI00334587.1	Heterogeneous nuclear ribonucleoprotein A/B
17	IPI00021187.1	RuvB-like 1
18	IPI00163782.1	Far upstream element binding protein 1
19	IPI00007074.1	Tyrosyl-tRNA synthetase
20	IPI00375441.1	Far upstream element (FUSE) binding protein 1
21	IPI00218342.6	C-1-tetrahydrofolate synthase, cytoplasmic
22	IPI00411623.1	Enabled protein homolog (MENA) similar to Avena
23	IPI00218342.6	C-1-tetrahydrofolate synthase, cytoplasmic
24	IPI00001661.1	Regulator of chromosome condensation (RCC1)
25	IPI00012079.1	Eukaryotic translation initiation factor 4B
26	IPI00009960.4	Mitochondrial inner membrane protein (Mitofilin)
27	IPI00411623.1	Enabled protein homolog (MENA) similar to Avena

Selected TOV-81D spots with undetectable TOV-112D matching spots		

28	IPI00022314.1	Superoxide dismutase mitochondrial precursor
29	IPI00018352.1	Ubiquitin carboxyl-terminal hydrolase isozyme L1
30	IPI00218694.1	Caldesmon
31	IPI00306959.5	Keratin, type II cytoskeletal 7 (Cytokeratin-7)
32	IPI00220709.3	Tropomyosin
33	IPI00171834.2	Keratin, type I cytoskeletal 19 (Cytokeratin 19)
34	IPI00418411.1	Keratin, type II cytoskeletal 8 (Cytokeratin 8)
35	IPI00333771.1	Caldesmon
36	IPI00007118.1	Endothelial plasminogen activator inhibitor) (PAI-1)
37	IPI00333771.1	Caldesmon
38	IPI00026663.1	Aldehyde dehydrogenase 6
39	IPI00008524.1	Poly(A)-binding protein 1 (PABP 1)
40	IPI00027341.1	Macrophage capping protein (Actin-regulatory protein CAP-G)
41	IPI00029111.1	Dihydropyrimidinase related protein-3
42	IPI00029111.1	Dihydropyrimidinase related protein-3
43	IPI00333771.1	Caldesmon
44	IPI00337495.1	Procollagen-lysine, 2-oxoglutarate 5-dioxygenase (Lysine hydroxylase) 2
45	IPI00337495.1	Procollagen-lysine, 2-oxoglutarate 5-dioxygenase (Lysine hydroxylase) 2
46	IPI00329536.1	Early endosome antigen 1 (Endosome-associated protein p162)

Selected spots differentially expressed with a minimum deregulation fold of 2.0		

47	IPI00219018.1	Glyceraldehyde-3-phosphate dehydrogenase
48	IPI00218918.1	Annexin I
49	IPI00003865.1	Heat shock cognate 71 kDa protein
50	IPI00018352.1	Ubiquitin carboxyl-terminal hydrolase isozyme L1
51	IPI00010796.1	Protein disulfide isomerase precursor (PDI)
52	IPI00022314.1	Superoxide dismutase mitochondrial precursor
53	IPI00025252.1	Protein disulfide-isomerase A3 precursor (Disulfide isomerase ER-60)
54	IPI00027350.1	Peroxiredoxin 2 (Thioredoxin-dependent peroxide reductase 1)
55	IPI00015262.5	Calponin H2
56	IPI00218694.1	Caldesmon
57	IPI00000877.1	150 kDa oxygen-regulated protein precursor
58	IPI00024911.1	Endoplasmic reticulum protein ERp29 precursor
59	IPI00021187.1	RUVB-like 1
60	IPI00218682.1	Prolyl 4-hydroxylase alpha-1 subunit precursor
61	IPI00007765.2	Stress-70 protein, mitochondrial precursor
62	IPI00219005.1	FK506-binding protein 4
63	IPI00216953.1	Lamin A/C
64	IPI00298363.2	Far upstream element binding protein 2
65	IPI00217056.1	Leprecan-like 2 protein

In addition to these observations, the present 2DE analysis underscores the modification of protein expression relative to redox regulation and detoxification pathways. Aldehyde dehydrogenase (Spot#1), a determinant protein in resistance to multiple chemotherapeutic agents through the inactivation of drugs into non cytotoxic metabolites is tremendously overexpressed in TOV-112D. However, proteins that are associated with the redox level of the cell such as superoxide dismutase (Spot#28), protein disulfide isomerase (PDI) (Spot#51) or peroxiredoxin (Spot#54) are generally found with more modest expression compared to TOV-81D. The adaptation of TOV-112D cells to hypoxia which was acquired from selection of transformed cells resistant to oxygen deprivation could explain that the expression of some proteins involved in the detoxification of oxygen reactive species. The adaptation of tumour cells to hypoxia and acidification of the environment is also known to promote survival over normal cells. When tumour cells face stressful conditions such as oxygen deprivation, the accumulation of misfolded proteins can induce the expression of chaperones and heat shock proteins, a family of overexpressed proteins systematically found in 2DE proteome analysis of ovarian cancer cells. Changes such as the overexpression of chaperones and hypoxia-related proteins may impact on the cellular behaviour of the TOV-112D cell line which is characterized by high proliferative index.

Recent advances in two-dimensional electrophoresis have greatly improved the reproducibility and analytical power of the technique but still suffer from several important limitations. The detection of low abundant spots and the unambiguous annotation of a quantitative change in a spot from which multiple protein identifications were made (protein co-migration) are still common issues. The protein dataset generated by the 2DE analysis is generally limited to high abundant proteins in contrast to the iTRAQ analysis that shows a wider panel of protein. However, 2DE remains a powerful technique to analyze differential protein expression because of its resolution of complex proteomes. Moreover, 2DE is a method of choice to indicate putative posttranslational modifications and protein isoforms. For example, the multiple high molecular weight proteins in a typical pearl necklace pattern identified as isoforms of collagen alpha (Spot#3) proteins suggests that this protein might be modified, presumably by glycosylation, a modification that can affect the cells' ability to adhere, migrate, and invade toward extracellular matrix components [46]. Interestingly, the collagen alpha gene has also been shown to be stimulated in hypoxia conditions [47], a physiological state likely relevant for TOV-112D cells.

### Validation of protein expression levels by immunological comparison of differentially expressed proteins identified by iTRAQ and 2DE analysis

**Figure 5:**
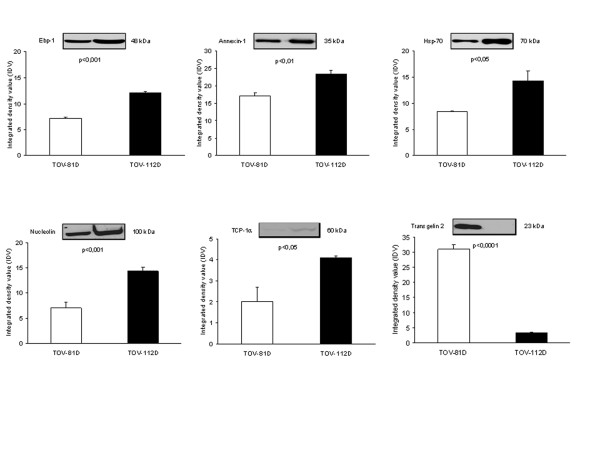
Western blot validation of selected proteins identified by iTRAQ and/or 2DE analysis. Equal amounts, 25 μg, of protein extracts from TOV-112D and TOV-81D cell lines were loaded onto a 12 % SDS-PAGE and processed for Western blotting with the indicated antibodies. Protein expression differences were quantified using a Chemilmager 4000 imaging system and AlphaEase software 3.3 (Alpha Innotech Corporation). The data are expressed as relative integrated density value (IDV). Each point represents the mean +/- SE from three independent experiments. Data were analysed by Student's unpaired *t *test. Representative blots for each analysis are depicted.

The deregulation of some protein candidates identified by iTRAQ or 2DE analysis has been validated by comparison with quantitative immunoblots. Western blot analysis has been performed to quantify protein expression of some target proteins. The quantitative Western blot analysis shown on Figure 5 for selected proteins follows the same deregulation trend as noticed with 2DE or iTRAQ analysis. Highly underexpressed proteins such as transgelin 2 are clearly validated by Western blot since there is a pronounced difference in signal intensity between the two cell lines. The statistical analysis of Western blot is consistent with 2DE and iTRAQ ratios evaluations. Converging data from 2DE, iTRAQ and Western blot analysis add further consistency and validation to our results.

## Conclusion

Understanding the mechanisms underlying EOC have been limited because little is known about the events of neoplastic transformation, mechanisms of invasion and metastatic dissemination. High-throughput differential protein expression analysis like the present study is aimed at the identification of those proteins that could collectively add valuable information. The proteome analysis presented here will be of use to the ovarian community to help discriminate candidates of interest. The rapid development of proteomics-based and protein microarray technologies brings new perspectives, such as systems biology, to make an integrative approach of both technologies and to ultimately give a global view of the cell behavior by monitoring interaction networks. By taking into account pathways, networks and mechanisms which all could dynamically be linked together, systems biology will broaden our view of the biological deregulations associated with cancer progression. The establishment of a repertoire of genes and proteins potentially involved in the cell's transition to transformed phenotype is the prerequisite to conceptualize ovarian cancer with an all-inclusive view.

## Methods

### Cell culture

Immortalized malignant epithelial ovarian tumour cell line TOV-112D and low malignancy TOV-81D cell lines were cultured (air/CO_2_, 19:1, 37°C) in medium consisting of 50:50 (v:v) medium 199: medium 105 (Sigma), supplemented with 10% foetal bovine serum (Hyclone). Penicillin (100 U/ml) and streptomycin (100 mg/ml) (Wisent) were added to culture media. When growth of cells reached approximately 75–80% confluency, cells were detached from the cell culture dishes using a Hanks Balanced Salt Solution containing 0.05% trypsin and 0.53 mM EDTA (Wisent). Cell pellets were washed with low salt PBS buffer (171 mM NaCl, 3.3 mM KCl,10 mM Na_2_PO_4_, 2 mM NaH_2_PO_4_) and stored at -80°C.

### Digestion and iTRAQ Labeling

100 μg of protein from each cell line was resuspended in 0.5 M triethyl ammonium bicarbonate (TEAB), and then reduced, alkylated, digested and labeled according to the standard protocol supplied by the manufacturer (Applied Biosystems iTRAQ™ Reagents – Chemistry Reference Guide, P/N 4351918A). iTRAQ results were generated from the analysis of three isobaric tags combinations. For the first combination, one sample from cell line TOV81D and one from cell line TOV112D were labeled with iTRAQ reagent 115 and 117, respectively. In a second analysis where two combinations were selected, two samples from each cell line were labeled. TOV81D was labeled with iTRAQ reagents 114 and 116, while TOV112D was labeled with iTRAQ reagents 115 and 117. Each cell line was therefore analyzed in triplicates and three ratios TOV112D/TOV81D were calculated (117/115 for analysis 1; 115/114 and 117/116 for analysis 2).

### Fractionation of tryptic peptides

#### Strong Cation Exchange (SCX) Fractionation

For the first and second experiments, tryptic peptides were fractionated using strong cation exchange (SCX) as follow. Combined labeled samples were brought up to 2 mL with buffer A (10 mM KH_2_PO_4_, pH 2.7, 25% ACN) and injected onto a Polysulfoethyl A, 5 μm, 300 Å, 4.6 mm id × 100 mm SCX column (Poly LC, Columbia, MD). Using a Vision Workstation (Applied Biosystems, Foster City, CA), the column was allowed to equilibrate for 20 minutes in buffer A before a gradient was applied: 0–35% B (10 mM KH_2_PO_4_, pH 2.7, 25% ACN, 500 mM KCl) in 30 minutes at a flow rate of 0.5 ml/min. Fractions were collected every minute after injection. For the LC-MS/MS analysis, the fractions are selected based on the UV elution profile, recorded at 215 nm. The selected fractions were then reduced in volume, to about 180 μL, in a Speed-Vac (Savant Instruments, Holbrook, NY) and transferred to autosampler vials (LC Packings, Amsterdam).

#### Fractionation of tryptic peptides by isoelectric focusing (IEF) on immobilized pH gradient

For the third experiment, separation of tryptic peptides according to their isoelectric point was used as a mean to obtain better protein coverage and more accurate quantitation for proteins that were identified with only one peptide by the two first analyses. Labeled peptides were lyophilized and resuspended in 315 μl of Milli-Q water containing 0.2 % carrier ampholytes (Bio-Lyte 3/10, Bio-Rad Laboratories). The resulting solution was used to rehydrate an 18-cm immobilized pH gradient gel strip (pH 5–8) for 10 hours at room temperature without any voltage applied. Peptides were focused by applying a voltage of 250V for 15 minutes, then 10 000V for 3 hours and finally 10 000 V for a total of 60 000 V•hour. Immediately after focusing, the strip was cut into 36 segments of 5mm. Gel pieces were transferred into a 96-well plate and peptides were eluted by first incubating the gel pieces for 15 minutes in 2% acetonitrile, 0.5% formic acid and then for 15 minutes in 50% acetonitrile, 0.5% formic acid. The extracted peptides were lyophilized using a SpeedVac and resuspended in 25 μl of 0.1% formic acid in water. 5 μl of this solution was used for LC-MS/MS analysis on a Q-TOF mass spectrometer (QSTAR-XL, Applied Biosystems) as described above.

#### LC-MS/MS analysis

Since the iTRAQ experiments were performed in two different proteomic facilities, the LC-MS/MS analyses were slightly different. For the first and second iTRAQ experiments, LC-MS/MS analysis was performed using an integrated Famos autosampler, SwitchosII switching pump, and UltiMate micro pump (LC Packings, Amsterdam) system with an Hybrid Quadrupole-TOF LC/MS/MS Mass Spectrometer (QSTAR Pulsar i, MDS Sciex, Concord, Ontario, Canada) equipped with a nano-electrospray ionization source (Proxeon, Odense, Denmark) and fitted with a 10-μm-ID fused-silica emitter tip (New Objective, Woburn, MA). About 25% of each fractions (40–50 μL) was injected onto a 300 μm id × 5 mm C18 PepMap guard column (5 μm, 100 Å, LC Packings, Amsterdam) and washed for 10 minutes with 95 % solvent A (water/ACN 98:2 (v/v), 0.05% FA) and 5 % solvent B (water/ACN 2:98 (v/v), 0.05% FA) at a flow rate of 100 μL/min. Chromatographic separation was achieved on a 75 μm id × 15 cm C18 PepMap Nano LC column (3 μm, 100 Å, LC Packings, Amsterdam) with a linear gradient from 5–60% solvent B in 40 minutes, at 200 nL/min. If the observed UV absorbance was greater than 0.1 for any fraction collected during the SCX, a 2-hours gradient was used to compensate for the larger amount of peptides in the fraction. MS data was acquired automatically using Analyst QS 1.0 software Service Pack 8 (MDS SCIEX, Concord, Ontario Canada). An information dependant acquisition (IDA) method, consisting of a 1 second TOFMS survey scan of mass range 400–1200 amu and two 2.5 second product ion scans of mass range 100–1500 amu, was used to fragment the two most intense peaks, above 20 counts, with charge state 2 to 5. Once an ion was selected for MS/MS fragmentation, it was put on an exclusion list for 180 seconds. A 6 amu window was used to prevent the peaks from the same isotopic cluster from being fragmented again.

For the third iTRAQ experiment, LC-MS/MS analysis was performed using the same LC system as described above coupled with a QSTAR xl mass spectrometer equipped with a nano-electrospray ionization source (MDS Sciex). 5 μl of each IEF fraction was injected, and peptides were trapped on a 300 μm ID × 5 mm C18 PepMap guard column (LC Packings) using a solution of 0.1% FA in water flowing at 15 μL/min. The peptide mixture was then separated on a 75 Am ID × 10 cm BioBasic C18 column (New Objective) at a flow rate of 200 nL/min. The chromatographic separation started at 98% buffer A (0.1% FA in water) and 2% buffer B (0.1% FA in acetonitrile) for 5 minutes. The gradient was then performed as follow: 2% to 25% B in 85 minute, 25% to 40% B in 10 min and 40% to 80% B in 5 min. Eluted peptides were electrosprayed through a 15-μm-ID fused-silica emitter tip (New objective) with an ion spray voltage of 2800 V. MS data was acquired automatically using Analyst QS 1.1 software (MDS SCIEX). An IDA method, consisting of a 1-second TOFMS survey scan of mass range 400–1600 amu and three 3-second product ion scans of mass range 100–2000 amu, was used to fragment the three most intense peaks, above 15 counts, with charge state 2 to 4. Fragmented target ions were dynamically excluded for 60 seconds with a 100 ppm mass tolerance.

### Data Analysis

Data files were processed using the ProQUANT software (version 1.0, Applied Biosystems, Foster City, CA) in Analyst using the IPI Human database (version 3.05, EBI) and the following parameters for searching. The MS and MS/MS tolerances were set to 0.20 Da, which take into account mass shift due to temperature variations. A tryptic digestion always precedes labelling with iTRAQ reagents and, therefore, ProQUANT always assumes the cleavage sites are lysine and arginine, and one missed cleavage was allowed. Methyl methanethiosulphonate (MMTS) modification of cysteines was used as a fixed modification. No variable modification or amino acids substitution were allowed. A threshold of 90 was set for peptide confidence. In order to reduce protein redundancy, ProGroup viewer (version 1.0.6, Applied Biosystems, Foster City, CA) was used to assemble and report the data.

For each peptide identification, ProQUANT computes the areas under the peaks at 114, 115, 116 and 117 Da, corresponding to the four iTRAQ reagents. A user-define denominator is used to calculate ratios (e.g. 115 was chosen as the denominator for the first analysis). A statistical determination of a significant over- or underexpression threshold has been performed on the iTRAQ dataset using the Statgraphics Centurion's (version 15.1.03, StatPoint, Herndon, VA), Generalized Logistic distribution to the ratios (TOV112D/TOV81D) for all the identified proteins. The proteins identified from each iTRAQ analysis were combined in a single table [see Additional file-1]. Average ratio (geometrical mean) and relative standard deviation (RSD) were calculated for each protein. Proteins with ratios above 2.5 and under 0.59 were then extracted from the list and manually validated. For higher protein identification and ratio confidence, only proteins with two, or more, validated unique peptides were conserved [see Additional file-2].

Mapping of the predominant biological processes of the differentially expressed proteins on the GO hierarchy was performed using BiNGO v1.0 [48] coupled to the visualization capacities of Cytoscape v2.4 [49]. GO annotations p-values were obtained by hypergeometric satistical test (cluster versus the whole annotation bank) and corrected using Benjamin and Hochberg False Discovery Rate included in the BiNGO software. GO database was obtained as of July 1st, 2007. Gominer [50] analysis reveal proteins distributions within selected GO categories.

### Protein extraction and sample preparation for two-dimensional gel electrophoresis

Cell pellets were resuspended in 2 ml of lysis buffer containing 50 mM Tris-HCl pH 8.0, 1 mM MgCl_2_, 0.1% Triton X-100 (Sigma), 250 U of Benzonase™ (Novagen) endonucleases (DNAse and RNAse) and Complete™ protease-inhibitor cocktail (according to Roche Diagnostics instructions). The cell extract was mixed for 5 minutes to achieve complete cell disruption and protein solubilization, and incubated for 30 minutes at 37°C to facilitate DNA and RNA degradation by the added endonucleases. The volume was adjusted to 3 ml with lysis buffer and the cell extract was mixed for another 5 minutes. Nine ml of ice-cold acetone (3 volumes) were added and the extract was kept at -30°C for 2 hours. The precipitate was centrifuged at 15000 × *g *for 15 minutes and the resulting protein pellet was washed twice with 10 ml of ice-cold acetone. The pellet was air-dried and resuspended in the Ready Prep-3™ 2D buffer (Bio-Rad) containing: 5 M urea, 2 M thiourea, 2% CHAPS, 2% SB 3–10, 40 mM Tris, 0.2% Bio-Lytes 3–10. Protein concentration was estimated with Bradford's protein assay (Bio-Rad).

### 2DE proteome analysis

350 μl of 2D buffer containing 250 μg of each protein sample were used for the rehydration of 18 cm immobilized non-linear pH 3–10 gradient (IPG) strips (Amersham Biosciences). A multi step IEF voltage program was applied to the strips on a Protean IEF cell (Bio-Rad): 50 V for 12 hours, 250 V for 15 minutes, 1000 V for 1 hour, from 1000 V to 8000 V in a 5 hours step and a final step of 60 000 V-hr at 8000 V. Strips were first reduced by incubation in the equilibration/reduction buffer (6 M Urea, 0.375 M Tris pH 8.8, 2% SDS, 20% glycerol, 2% (w/v) DTT (Sigma)) and then alkylated in the same buffer but containing 2.5% (w/v) iodoacetamide (Sigma) instead of DTT. The second dimension was accomplished by running the strips on 1.5 mm-thick SDS/10%-(w/v)-polyacrylamide gels using the Protean II XL Multi-Cell (Bio-Rad). The electrophoresis unit was cooled at 20°C with a water circulation system and 40 mA/gel constant amperage was applied to the system. Gels were stained for 18 hours with SYPRO Ruby protein stain (Bio-Rad) according to manufacturer's instructions.

### 2DE Image analysis

Image acquisition was made using the CCD-based multi-wavelength fluoro-imager PROXPress™ proteomic imaging system (Perkin Elmer) at 100 μm resolution. A flat field fluorescence correction was applied for SYPRO Ruby fluorescence specifications: excitation and emission filters respectively at 480/30 and 620/30 nm. Image analysis and spot detection was accomplished with PDQuest™ 2-D analysis software version 7.3 (Bio-Rad) using Gaussian spot modeling. For quantitative spot comparison across gels, matchsets of three replicates of TOV-81D and TOV-112D 2D-gels were created. Automated and manual spot matching has been performed. An analysis set of proteins have been created to identify spots that are statistically significant. This analysis set of differentially expressed proteins is composed of spots unique to TOV-81D or TOV-112D and protein spots shared by the two replicate groups with a quantity variation threshold of 2.0. Replicate groups of TOV-81D and TOV-112D allowed the estimate of average quantities of their protein spots. Student's t-test statistical analysis with 95% significance level has been applied to the replicate groups. 155 proteins that match the threshold and statistical analysis criteria were selected for automated in-gel excision using a Spot Cutter system (Bio-Rad).

### Immunoblotting

For immunoblotting, cells were washed with ice-cold phosphate-buffered saline and lysed in 1X lysis buffer containing 50 mM Tris-HCl pH 8.0, 1 mM MgCl_2_, 0.1% Triton X-100 (Sigma), 250 U of Benzonase™ (Novagen, San Diego, CA) endonucleases (DNAse and RNAse) and Complete™ protease-inhibitor cocktail (according to Roche Diagnostics instructions). The cell extracts were mixed for 5 minutes to achieve complete cell disruption and protein solubilization, and incubated for 30 minutes at 37°C to facilitate DNA and RNA degradation by the added endonucleases. The protein lysates were centrifuged at 15000 g for 5 minutes and the protein concentration in the supernatant was estimated with Bradford's protein assay (Bio-Rad, Missisauga, Canada). Protein extracts (25 μg) were separated on SDS-PAGE and then transferred onto a PVDF membrane (Millipore, Bedford, MA). After incubating 1 hour with blocking solution (PBS-T containing 5% non-fat milk), the membrane was probed overnight, at room temperature with shaking, by primary antibodies to Annexin 1, rabbit polyclonal antibody (1:10 000) (Zymed Laboratories, South San Francisco, CA); Hsp-70, mouse monoclonal antibody (1:5000) and TCP-1α, mouse monoclonal antibody (1:1000) (Calbiochem, San Diego, CA); Ebp1, rabbit polyclonal antibody (1:5000) (Oncogene Research Products, San Diego, CA); Transgelin (SM22α), rabbit polyclonal antibody (1:10 000) (Abcam, Cambridge, MA) and nucleolin, mouse monoclonal antibody (1:2000) (Upstate Cell Signaling, Lake Placid, NY). After washing with PBS-T, species-specific horseradish peroxidase-conjugated secondary antibody was added for 1 hour at room temperature. Signals were detected with Western Lightning™ Chemiluminescence reagent plus kit (Perkin Elmer, Boston, MA).

## Authors' contributions

JPG and GGP conceived of the study, participated in its design and coordination and drafted the manuscript. JPG performed two-dimensional gel electrophoresis and image analysis. PG, GM and EW carried out the mass spectrometric analysis, protein identifications and statistical analysis. CE and MEB participated in the design of the study and performed cell culture, sample preparation, data analysis and validation. AMMM initiated this study, has been involved as an expert in ovarian cancer and in critically revising the manuscript. GGP and AMMM provided direction and funding of this project. AD and MI were charged of bioinformatics support and protein database maintenance.

## Supplementary Material

Additional file 1 Complete iTRAQ protein identification data. This list represent every protein identified by MS analysis for the three iTRAQ experiments, including peptide sequences and statistical information. Click here for file

Additional file 2 Statistically significant differential expression ratios that meet the 90% confidence criteria. Listing of differentially expressed proteins between TOV-81D and TOV-112D cell lines taken into consideration for this study. Click here for file

## References

[B1] Gagne JP, Gagne P, Hunter JM, Bonicalzi ME, Lemay JF, Kelly I, Le Page C, Provencher D, Mes-Masson AM, Droit A, Bourgais D, Poirier GG (2005). Proteome profiling of human epithelial ovarian cancer cell line TOV-112D. Mol Cell Biochem.

[B2] Provencher DM, Lounis H, Champoux L, Tetrault M, Manderson EN, Wang JC, Eydoux P, Savoie R, Tonin PN, Mes-Masson AM (2000). Characterization of four novel epithelial ovarian cancer cell lines. In Vitro Cell Dev Biol Anim.

[B3] Tonin PN, Hudson TJ, Rodier F, Bossolasco M, Lee PD, Novak J, Manderson EN, Provencher D, Mes-Masson AM (2001). Microarray analysis of gene expression mirrors the biology of an ovarian cancer model. Oncogene.

[B4] Presneau N, Mes-Masson AM, Ge B, Provencher D, Hudson TJ, Tonin PN (2003). Patterns of expression of chromosome 17 genes in primary cultures of normal ovarian surface epithelia and epithelial ovarian cancer cell lines. Oncogene.

[B5] Benoit MH, Hudson TJ, Maire G, Squire JA, Arcand SL, Provencher D, Mes-Masson AM, Tonin PN (2007). Global analysis of chromosome X gene expression in primary cultures of normal ovarian surface epithelial cells and epithelial ovarian cancer cell lines. Int J Oncol.

[B6] Schwartz DR, Kardia SL, Shedden KA, Kuick R, Michailidis G, Taylor JM, Misek DE, Wu R, Zhai Y, Darrah DM, Reed H, Ellenson LH, Giordano TJ, Fearon ER, Hanash SM, Cho KR (2002). Gene expression in ovarian cancer reflects both morphology and biological behavior, distinguishing clear cell from other poor-prognosis ovarian carcinomas. Cancer Res.

[B7] Gilks CB (2004). Subclassification of ovarian surface epithelial tumors based on correlation of histologic and molecular pathologic data. Int J Gynecol Pathol.

[B8] Manderson EN, Mes-Masson AM, Novak J, Lee PD, Provencher D, Hudson TJ, Tonin PN (2002). Expression profiles of 290 ESTs mapped to chromosome 3 in human epithelial ovarian cancer cell lines using DNA expression oligonucleotide microarrays. Genome Res.

[B9] Manderson EN, Presneau N, Provencher D, Mes-Masson AM, Tonin PN (2002). Comparative analysis of loss of heterozygosity of specific chromosome 3, 13, 17, and X loci and TP53 mutations in human epithelial ovarian cancer. Mol Carcinog.

[B10] Presneau N, Dewar K, Forgetta V, Provencher D, Mes-Masson AM, Tonin PN (2005). Loss of heterozygosity and transcriptome analyses of a 1.2 Mb candidate ovarian cancer tumor suppressor locus region at 17q25.1-q25.2. Mol Carcinog.

[B11] Wu WW, Wang G, Baek SJ, Shen RF (2006). Comparative study of three proteomic quantitative methods, DIGE, cICAT, and iTRAQ, using 2D gel- or LC-MALDI TOF/TOF. J Proteome Res.

[B12] Ross PL, Huang YN, Marchese JN, Williamson B, Parker K, Hattan S, Khainovski N, Pillai S, Dey S, Daniels S, Purkayastha S, Juhasz P, Martin S, Bartlet-Jones M, He F, Jacobson A, Pappin DJ (2004). Multiplexed protein quantitation in Saccharomyces cerevisiae using amine-reactive isobaric tagging reagents. Mol Cell Proteomics.

[B13] Ashburner M, Ball CA, Blake JA, Botstein D, Butler H, Cherry JM, Davis AP, Dolinski K, Dwight SS, Eppig JT, Harris MA, Hill DP, Issel-Tarver L, Kasarskis A, Lewis S, Matese JC, Richardson JE, Ringwald M, Rubin GM, Sherlock G (2000). Gene ontology: tool for the unification of biology. The Gene Ontology Consortium. Nat Genet.

[B14] Gu L, Shigemasa K, Ohama K (2004). Increased expression of IGF II mRNA-binding protein 1 mRNA is associated with an advanced clinical stage and poor prognosis in patients with ovarian cancer. Int J Oncol.

[B15] Dimitriadis E, Trangas T, Milatos S, Foukas PG, Gioulbasanis I, Courtis N, Nielsen FC, Pandis N, Dafni U, Bardi G, Ioannidis P (2007). Expression of oncofetal RNA-binding protein CRD-BP/IMP1 predicts clinical outcome in colon cancer. Int J Cancer.

[B16] Doyle GA, Betz NA, Leeds PF, Fleisig AJ, Prokipcak RD, Ross J (1998). The c-myc coding region determinant-binding protein: a member of a family of KH domain RNA-binding proteins. Nucleic Acids Res.

[B17] Adhikary S, Eilers M (2005). Transcriptional regulation and transformation by Myc proteins. Nat Rev Mol Cell Biol.

[B18] Bianchi ME, Agresti A (2005). HMG proteins: dynamic players in gene regulation and differentiation. Curr Opin Genet Dev.

[B19] Tallini G, Dal Cin P (1999). HMGI(Y) and HMGI-C dysregulation: a common occurrence in human tumors. Adv Anat Pathol.

[B20] Masciullo V, Baldassarre G, Pentimalli F, Berlingieri MT, Boccia A, Chiappetta G, Palazzo J, Manfioletti G, Giancotti V, Viglietto G, Scambia G, Fusco A (2003). HMGA1 protein over-expression is a frequent feature of epithelial ovarian carcinomas. Carcinogenesis.

[B21] Lancaster JM, Dressman HK, Clarke JP, Sayer RA, Martino MA, Cragun JM, Henriott AH, Gray J, Sutphen R, Elahi A, Whitaker RS, West M, Marks JR, Nevins JR, Berchuck A (2006). Identification of genes associated with ovarian cancer metastasis using microarray expression analysis. Int J Gynecol Cancer.

[B22] Lee BC, Cha K, Avraham S, Avraham HK (2004). Microarray analysis of differentially expressed genes associated with human ovarian cancer. Int J Oncol.

[B23] Anand N, Murthy S, Amann G, Wernick M, Porter LA, Cukier IH, Collins C, Gray JW, Diebold J, Demetrick DJ, Lee JM (2002). Protein elongation factor EEF1A2 is a putative oncogene in ovarian cancer. Nat Genet.

[B24] Tomlinson VA, Newbery HJ, Wray NR, Jackson J, Larionov A, Miller WR, Dixon JM, Abbott CM (2005). Translation elongation factor eEF1A2 is a potential oncoprotein that is overexpressed in two-thirds of breast tumours. BMC Cancer.

[B25] Kauppila S, Saarela J, Stenback F, Risteli J, Kauppila A, Risteli L (1996). Expression of mRNAs for type I and type III procollagens in serous ovarian cystadenomas and cystadenocarcinomas. Am J Pathol.

[B26] Shields JM, Rogers-Graham K, Der CJ (2002). Loss of transgelin in breast and colon tumors and in RIE-1 cells by Ras deregulation of gene expression through Raf-independent pathways. J Biol Chem.

[B27] Yeo M, Kim DK, Park HJ, Oh TY, Kim JH, Cho SW, Paik YK, Hahm KB (2006). Loss of transgelin in repeated bouts of ulcerative colitis-induced colon carcinogenesis. Proteomics.

[B28] Chen H, Wang M, Wang XY, Gao S, Wang J, Guan XM (2005). Identification of differential genes in ovarian cancer using representational difference analysis of cDNA. Chin Med Sci J.

[B29] Ren B, Yee KO, Lawler J, Khosravi-Far R (2006). Regulation of tumor angiogenesis by thrombospondin-1. Biochim Biophys Acta.

[B30] Lawler J (2002). Thrombospondin-1 as an endogenous inhibitor of angiogenesis and tumor growth. J Cell Mol Med.

[B31] Sid B, Sartelet H, Bellon G, El Btaouri H, Rath G, Delorme N, Haye B, Martiny L (2004). Thrombospondin 1: a multifunctional protein implicated in the regulation of tumor growth. Crit Rev Oncol Hematol.

[B32] Alvarez AA, Axelrod JR, Whitaker RS, Isner PD, Bentley RC, Dodge RK, Rodriguez GC (2001). Thrombospondin-1 expression in epithelial ovarian carcinoma: association with p53 status, tumor angiogenesis, and survival in platinum-treated patients. Gynecol Oncol.

[B33] Zietarska M, Maugard CM, Filali-Mouhim A, Alam-Fahmy M, Tonin PN, Provencher DM, Mes-Masson AM (2007). Molecular description of a 3D in vitro model for the study of epithelial ovarian cancer (EOC). Mol Carcinog.

[B34] Hirtenlehne K, Pec M, Kubista E, Singer CF (2002). Extracellular matrix proteins influence phenotype and cytokine expression in human breast cancer cell lines. Eur Cytokine Netw.

[B35] Guan G, Dai P, Shechter I (2003). cDNA cloning and gene expression analysis of human myo-inositol 1-phosphate synthase. Arch Biochem Biophys.

[B36] Miyasaka KY, Kida YS, Sato T, Minami M, Ogura T (2007). Csrp1 regulates dynamic cell movements of the mesendoderm and cardiac mesoderm through interactions with Dishevelled and Diversin. Proc Natl Acad Sci U S A.

[B37] Critchley DR, Holt MR, Barry ST, Priddle H, Hemmings L, Norman J (1999). Integrin-mediated cell adhesion: the cytoskeletal connection. Biochem Soc Symp.

[B38] Hashimoto Y, Skacel M, Adams JC (2005). Roles of fascin in human carcinoma motility and signaling: prospects for a novel biomarker?. Int J Biochem Cell Biol.

[B39] Yamada KM, Pankov R, Cukierman E (2003). Dimensions and dynamics in integrin function. Braz J Med Biol Res.

[B40] Choong PF, Nadesapillai AP (2003). Urokinase plasminogen activator system: a multifunctional role in tumor progression and metastasis. Clin Orthop Relat Res.

[B41] Koensgen D, Mustea A, Denkert C, Sun PM, Lichtenegger W, Sehouli J (2006). Overexpression of the plasminogen activator inhibitor type-1 in epithelial ovarian cancer. Anticancer Res.

[B42] Vasiliou V, Nebert DW (2005). Analysis and update of the human aldehyde dehydrogenase (ALDH) gene family. Hum Genomics.

[B43] Kauppila S, Stenback F, Risteli J, Jukkola A, Risteli L (1998). Aberrant type I and type III collagen gene expression in human breast cancer in vivo. J Pathol.

[B44] Tapper J, Kettunen E, El-Rifai W, Seppala M, Andersson LC, Knuutila S (2001). Changes in gene expression during progression of ovarian carcinoma. Cancer Genet Cytogenet.

[B45] Gerke V, Creutz CE, Moss SE (2005). Annexins: linking Ca2+ signalling to membrane dynamics. Nat Rev Mol Cell Biol.

[B46] Casey RC, Oegema TR, Skubitz KM, Pambuccian SE, Grindle SM, Skubitz AP (2003). Cell membrane glycosylation mediates the adhesion, migration, and invasion of ovarian carcinoma cells. Clin Exp Metastasis.

[B47] Falanga V, Zhou L, Yufit T (2002). Low oxygen tension stimulates collagen synthesis and COL1A1 transcription through the action of TGF-beta1. J Cell Physiol.

[B48] Maere S, Heymans K, Kuiper M (2005). BiNGO: a Cytoscape plugin to assess overrepresentation of gene ontology categories in biological networks. Bioinformatics.

[B49] Shannon P, Markiel A, Ozier O, Baliga NS, Wang JT, Ramage D, Amin N, Schwikowski B, Ideker T (2003). Cytoscape: a software environment for integrated models of biomolecular interaction networks. Genome Res.

[B50] Zeeberg BR, Feng W, Wang G, Wang MD, Fojo AT, Sunshine M, Narasimhan S, Kane DW, Reinhold WC, Lababidi S, Bussey KJ, Riss J, Barrett JC, Weinstein JN (2003). GoMiner: a resource for biological interpretation of genomic and proteomic data. Genome Biol.

